# Trajectories of working hours in later careers and their association with social and health-related factors: a follow-up study

**DOI:** 10.1093/eurpub/ckab179

**Published:** 2021-10-05

**Authors:** Johanna Suur-Uski, Olli Pietiläinen, Ossi Rahkonen, Tea Lallukka

**Affiliations:** 1 Department of Public Health, University of Helsinki, Helsinki, Finland; 2 Finnish Institute of Occupational Health, Helsinki, Finland

## Abstract

**Background:**

The aim was to identify working hours’ trajectories in later work careers over a follow-up of 15–17 years and to examine their association with social factors and health.

**Methods:**

A subsample from the Helsinki Health Study was extracted comprising employees of the City of Helsinki, Finland. Growth mixture modelling was used to identify different working hour trajectories. Age, gender, occupational class, marital status, health behaviour, physical and mental functioning and current pain were associated with trajectory membership. Relative risks (RRs) and their 95% confidence intervals (CIs) were estimated.

**Results:**

A two-trajectory model was selected: ‘Stable regular working hours’ (90%) and ‘Shorter and varying working hours’ (10%). Women (RR 1.40, 95% CI 1.09–1.78), the oldest employees (RR 2.71, 95% CI 2.06–3.57), managers and professionals (RR 1.56, 95% CI 1.20–2.02), those reporting non-drinker (RR 1.66, 95% CI 1.32–2.10), those reporting sleeping more than 8 h per night (RR 1.74 95% CI 1.25–2.42) and those reporting poor mental functioning (RR 1.39 95% CI 1.15–1.68) had higher likelihood of belonging to the trajectory ‘Shorter and varying working hours’. There were no differences between the trajectories in marital status, smoking, body mass index, current pain or physical functioning. However, routine non-manual workers (RR 0.74, 95% CI 0.55–0.98), and semi-professionals (RR 0.70, 95% CI 0.50–0.96) had lower likelihood of belonging to this trajectory.

**Conclusions:**

Trajectories of working hours in later work career differ by age, gender and occupational class but also by health behaviours and mental health functioning.

## Introduction

Many countries are experiencing population ageing.^1^ During the past decades multiple European countries have increased the statutory retirement age aiming to raise the proportion of the employed population. It is projected that in the European Union (EU), the share of all employees aged 55–64 years will reach 20% in the 2020s.^2^ Nowadays, those aged above 54 years are less employed compared to their younger counterparts even if positive trend is evident; in 2018, the employment rate of 55- to 64-year-olds was 57% when in 2006 it was 43%.^3^ Similarly, in the EU, many countries have implemented policies facilitating working time reductions for the older employees.^4^ According to Organisation for Economic Co-operation and Development statistics employees above 54 years work shorter hours compared to 25- to 54-year-old employees.^5^ In light of these changes, more in-depth understanding is needed about who and with what characteristics work until their normal retirement age and how their working hours develop in later careers.

Previous research has focussed on those exiting workforce prematurely. Some studies associate exiting labour force prematurely with poor health and lower socio-economic position^6^ and longer working life with good health^7^ and higher socio-economic position^8^ but other studies find no association between early retirement and health. Poor health does not always mean exit from paid employment and altering weekly working hours and other workplace modifications is an occupational health care tool by which employees’ work ability can be maintained.^9^ In Finland, women work shorter hours and more often part-time, but men and women report equally often working overtime. Multiple adverse health effects have been linked to long working hours^10–15^ but few studies have examined working hours in later career.^16^ Additionally, shorter weekly working hours have received less attention.^17^

Most previous studies on working hours are variable-oriented, assuming a homogenous sample and focussing on associations between variables. Growth mixture modelling (GMM) models longitudinal data by detecting patterns within a heterogeneous data and dividing people into qualitatively different (latent) groups without prior assumptions.^18^ To our knowledge, there are no previous studies that use trajectory modelling in examining and identifying distinct developmental patterns of working hours from midlife towards retirement.^16^ Our aim is to first identify trajectories in working hours in midlife and later careers, and second to examine social- and health-related factors associated with the trajectories’ memberships.

## Methods

Data were derived from the Helsinki Health Study (HHS), a cohort of midlife and ageing employees of the City of Helsinki, Finland.^19^

The questionnaires were mailed to employees turning 40, 45, 50, 55 and 60 in three consecutive years 2000–02. At baseline (Phase 1), the sample consisted of 8960 employees (response rate 67%). Approximately 80% of the participants were women corresponding with the share of women in the City of Helsinki and elsewhere in the public sector in Finland. The follow-up questionnaires were mailed in 2007, 2012 and 2017 to all respondents of the baseline survey. Response rates for the follow-up questionnaires in 2007 (Pase 2), 2012 (Phase 3) and 2017 (Phase 4) were 83%, 79% and 82%, respectively.

### Study population

We included participants, who on at least one of the phases reported both: (i) having at least one working hour per week and (ii) being employed full-time or part-time. At Phase 1, all participants were employed and reported working hours. At each follow-up, those who did not meet these criteria were excluded from the analysis at that phase, but were included in later phases if meeting the criteria at that phase. The total number of included participants at each phase was: 8552 at Phase 1, 5060 at Phase 2, 3367 at Phase 3 and 2448 at Phase 4. Main reason for not to be included was retirement (and attrition).

### Working hours

Working hours were measured as follows: in Phases 1–3, options for working hours were: 1–10, 11–29, 30–40, 41–50 and over 50 h per week. At Phase 4, participants could additionally report working 0 h per week but the participants who reported being employed but working 0 h per week (*n* = 8) were omitted from the analyses. Most commonly reported category was 30–40 (73%). Reporting 1–10 working hours per week was rare (3%).

### Covariates

Age, gender, occupational class, marital status, current smoking, alcohol consumption, sleeping hours, body mass index (BMI), current pain and physical and mental functioning were used as covariates. Age and gender were derived from Phase 1. Occupational class was derived from the personnel register for those with an informed consent to link their survey data to register data (78%), and completed from the questionnaires for the rest. Occupational class was classified into four categories: professionals and managers, semi-professionals, routine non-manual employees and manual workers. Mode was used as during the follow-up participants could move to a different occupational class. Regarding all other categorical covariates, we used the mode derived from participants’ answers across all the phases (1–4). Marital status was dichotomized as cohabiting (married/cohabiting) or not-cohabiting (divorced/widowed/single). Current smoking was reported as ‘yes’ or ‘no’. Reported alcohol consumption was classified into three categories: non-drinkers, non-binge drinkers and binge drinkers, which was defined as drinking 6 or more units in one occasion. Mean BMI (from self-reported weight and height) was computed across all the study phases (1–4) and was classified into three categories: healthy weight (<25.0 kg/m^2^), overweight (25.0–30.0 kg/m^2^) and obesity (>30.0 kg/m^2^). Reported sleeping hours were classified into three categories: <7 h per night, 7–8 h per night and over 8 h per night. Current pain was based on the question: ‘Do you have pain at the moment?’ with response alternatives ‘yes’ or ‘no’. Health outcomes were physical and mental health functioning measured by the Short Form 36 questionnaire.^20^ A Finnish validated translation was used.^21^ Continuous physical and mental component summary scores were calculated. Higher score indicates better functioning. The lowest quartile was assigned ‘poor functioning’. Household net income was derived from the baseline questionnaire and analyzed separately^22^ (Supplementary table S1).

### Statistical analyses

We modelled the development of working hours using trajectory modelling. The trajectories were estimated using GMM, a subcategory of finite mixture modelling. GMM studies longitudinal data by detecting patterns within a heterogeneous study population and by those patterns classifying individuals into qualitatively different latent trajectory groups.^18^^,^^23^GMM accounts for within class variability between subjects as random effects are used to account for individual differences within latent classes.^24^ With GMM measurements can vary from one subject to another allowing to for example include participants with intermittent missing data or dropout.^25^ The analyses were computed with the R 3.5.2 software^26^ using the package ‘lcmm’.^25^ The optimal number of trajectory groups was selected based on the following criteria: Bayesian information criteria, posterior probabilities of trajectory group membership, Akaike information criteria, identifiability of the distinct classes and with a preference to models that produced trajectory groups with no fewer than 5% of the participants.^27^ Model parameters are presented in the Supplementary material. Participants were assigned to the trajectory groups to which they had the highest probability of belonging to.^27^ Mean posterior probabilities were 99.7% in the trajectory ‘Stable regular working hours’ and 94.5% in ‘Shorter and varying working hours’, indicating a good model fit, high reliability and a low likelihood of classification error.

We first analyzed men and women together and then separately. The model with only women produced trajectories similar to the combined model (data not shown). The model with only men produced three trajectories, with a small third trajectory in which participants reported working hours over 40 h per week (data not shown). The model with only men was too small to be included in further analyses due to the small number of participant assigned to each trajectory.

The chi2 test was applied for categorical variables. We computed a relative risk (RR) and their 95% confidence intervals (95% CIs) for belonging to ‘Shorter and varying working hours’ trajectory using log-binomial regression, which is a type of generalized linear model using a log link function. In the first model, only age and gender were used. The second model included gender and age with each covariate independently. In the final model age, gender and all the other explanatory variables were simultaneously adjusted for. Household income was not included in the model as municipal employees’ income is closely linked to their occupational status.

## Results

### Descriptive statistics

A third of the participants were routine non-manual workers and another third were managers or professionals (table 1). Manual workers were the smallest occupational class (16%). Two-thirds were married or cohabiting. One-fifth reported smoking and half binge drinking. More than third (38%) were overweight and 17% obese. Daily sleep <7 h per night was reported by one-fifth and daily sleep over 8 h by 3%. The lowest quartile was assigned poor mental, and physical functioning and current pain was reported by 42%. Women more often belonged to the lower household income quartiles and men to the higher (Supplementary table S1).

**Table 1:** Baseline characteristics of the study population and characteristics of the trajectories. Trajectory 1= ‘Stable regular working hours’; Trajectory 2= ‘Shorter and varying working hours’

		Trajectories		
	Study population	Trajectory 1:	Trajectory 2:	*P*-value for chi^2^
		stable normal working hours	shorter and varying working hours	
*N* (%)	8792 (100%)	7941 (90%)	851 (10%)	
Women (%)	7036 (80%)	6317 (80%)	719 (84%)	<0.001
Age (%)				
40	1786 (20%)	1654 (21%)	132 (15.5%)	
45	1888 (21%)	1746 (22%)	142 (16.7%)	
50	1917 (22%)	1773 (22%)	144 (16.9%)	
55	2175 (25%)	1980 (25%)	195 (22.9%)	
60	1026 (12%)	788 (10%)	238 (28.0%)	
	8792 (100%)	7941 (100%)	851 (100%)	<0.001
Occupational class				
Professionals and managers	2638 (30%)	2233 (28.4%)	405 (48%)	
Semi-professionals	1768 (20%)	1651 (21.0%)	117 (14%)	
Routine non-manual workers	2901 (33%)	2693 (34.2%)	208 (25%)	
Manual workers	1401 (16%)	1289 (16.4%)	112 (13%)	
	8708 (100%)	7866 (100%)	842 (100%)	<0.001
Marital status				
Cohabiting	5966 (68%)	5395 (68%)	571 (67%)	
Non-cohabiting	2822 (32%)	2542 (32%)	280 (33%)	
	8788 (100%)	7937 (100%)	851 (100%)	0.63
Health behaviour				
Smoker	1773 (20%)	1640 (21%)	133 (16%)	
Non-smoker	6997 (80%)	6280 (79%)	717 (84%)	
	8770 (100%)	7920 (100%)	850 (100%)	<0.001
Alcohol consumption				
Non-drinker	699 (8%)	597 (8%)	102 (12%)	
Non-binge drinker	3648 (42%)	3236 (41%)	412 (48%)	
Binge drinker	4430 (50%)	4094 (52%)	336 (40%)	
	8777 (100%)	7927 (100%)	850 (100%)	<0.001
BMI				
Healthy weight, BMI < 25	2390 (45%)	2141 (45%)	249 (49%)	
Overweight	2001 (38%)	1828 (38%)	173 (34%)	
Obesity	869 (17%)	786 (17%)	83 (16%)	
	5260 (100%)	4755 (100%)	505 (100%)	0.14
Sleeping hours				
<7 h	1893 (22%)	1733 (22%)	160 (19%)	
7–8 h	6592 (75%)	5954 (75%)	638 (75%)	
9 h or more	284 (3%)	235 (3%)	49 (6%)	
	8769 (100%)	7922 (100%)	847 (100%)	<0.001
Physical functioning				
Good	6741 (77%)	6126 (77%)	615 (73%)	
Poor	2008 (23%)	1781 (23%)	227 (27%)	
	8749 (100%)	7907 (100%)	842 (100%)	0.004
Mental functioning				
Good	6798 (78%)	6167 (78%)	622 (74%)	
Poor	1960 (22%)	1740 (22%)	220 (26%)	
	8758 (100%)	7907 (100%)	842 (100%)	0.007
Current pain				
Yes	3712 (42%)	3356 (42%)	356 (42%)	
No	5048 (58%)	4558 (58%)	490 (56%)	
	8769 (100%)	7914 (100%)	855 (100%)	0.88

### Trajectories

**Figure 1:**
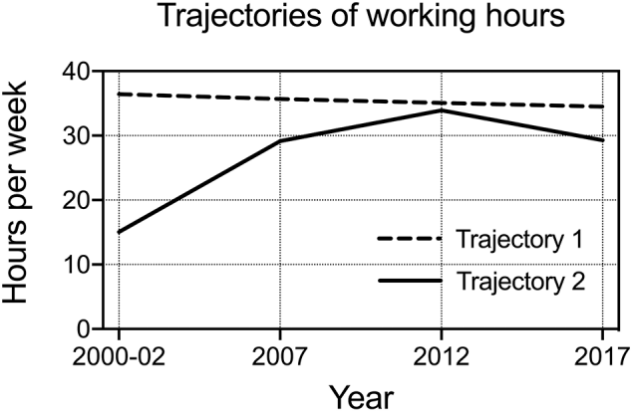
Trajectories of working hours among midlife and ageing employees. Trajectory 1 = ‘Stable regular working hours’ (90%); Trajectory 2 = ‘Shorter and varying working hours’ (10%)

Based on model statistics (Supplementary material) and interpretation, we selected a model with two working hour trajectories, ‘Stable regular working hours’ and ‘Shorter and varying working hours’ (figure 1).

The first trajectory, ‘Stable regular working hours’, included 90% of the study population (table 1) and consisted of employees reporting working hours at around 35 h per week. In this trajectory, the weekly working hours only declined slightly, from 37 h per week to 35 h during the follow-up.

The second trajectory group, ‘Shorter and varying working hours’, consisted of individuals who in contrast to ‘Stable regular working hours’ worked shorter hours throughout the follow-up. In this trajectory, working hours started at Phase 1 from 15 h per week, increased to 34 h at Phase 3, and declined to <30 h per week at Phase 4. In both trajectory groups, the majority of the employees worked full-time jobs and daytime work was reported by 85% in the trajectory ‘Shorter and varying working hours’ and by 78% in ‘Stable working hours’. Shift work was reported more often in the trajectory ‘Stable working hours’ with 19% compared to 9% in the ‘Shorter and varying working hours’ (*P* < 0.001) (data not shown).

Members of the trajectory ‘Shorter and varying working hours’ were more often women and from the oldest age group (aged 60 at Phase 1) compared to the trajectory ‘Stable regular working hours’. Half of the members of the trajectory ‘Shorter and varying working hours’ belonged to professionals and managers while the corresponding proportion was 28% in ‘Stable regular working hours’ (*P* < 0.001). Trajectory ‘Stable regular working hours’ had a higher percentage of the routine employees (34% vs. 25%), semi-professionals (21% vs. 14%) and manual workers (16% vs. 13%) (*P* < 0.001). There were no differences by marital status (*P* = 0.63).

Reporting smoking (21% vs. 16%, *P* < 0.001) and binge drinking (52% vs. 40%, *P* < 0.001) was more common in trajectory ‘Stable regular working hours’, whereas reporting non-drinker was more common (12% vs. 8%, *P* < 0.001) in ‘Shorter and varying working hours’. There were no differences by BMI (*P* = 0.14).

Reporting sleeping <7 h was more common (22% vs. 19%) and reporting sleeping >8 h more rare (3% vs. 6%) in trajectory ‘Stable regular working hours’ (*P* < 0.001). Poor physical functioning was reported by 27% in ‘Shorter and varying working hours’ vs. 23% (*P* = 0.004) and poor mental functioning by 26% vs. 22% (*P* = 0.007). Current pain was reported by 42% in both trajectories.

### Social and health-related factors

In Model 1 (table 2), women (RR 1.41, 95% CI 1.19–1.68) compared to men and those in the oldest age group (RR 3.18, 95% CI 2.61–388) compared to the youngest age group were more likely to belong to the trajectory of ‘Shorter and varying working hours’ (table 2).

**Table 2:** Social and health-related factors risk ratio of belonging to Trajectory 2 compared to Trajectory 1 [log-binomial regression model, risk ratio (RR for belonging to Trajectory 2 compared to Trajectory 1 and 95% CI)]

	**Model 1** ^a^	**Model 2** ^a^	**Model 3** ^a^
		RR (95% CI)^b^	
Women	1.41 (1.19–1.68)^c^		1.40 (1.09–1.78)^c^
Age (ref: 40 years at Phase 1)			
Age 45	1.02 (0.81–1.28)		1.03 (0.75–1.40)
Age 50	1.02 (0.81–1.28)		1.03 (0.76–1.39)
Age 55	1.23 (0.99–1.52)		1.12 (0.84–1.49)
Age 60	3.18 (2.61–3.88)^c^		2.71 (2.06–3.57)^c^
Occupational class (ref: manual workers)			
Professionals and managers		1.87 (1.54–2.27)^c^	1.56 (1.20–2.02)^c^
Routine non-manual workers		0.86 (0.69–1.07)	0.74 (0.55–0.98)^c^
Semi-professionals		0.83 (0.65–1.07)	0.70 (0.50–0.96)^c^
Marital status (ref: cohabiting)			
Non-cohabiting		0.96 (0.84–1.10)	0.99 (0.83–1.18)
Health behaviour			
Smoker (ref: non-smoker)		0.82 (0.69–0.98)^c^	0.88 (0.67–1.15)
Drinking habits (ref: non-binge drinker)			
Binge drinker		0.82 (0.71–0.95)^c^	0.82 (0.68–0.999)^c^
Non-drinker		1.28 (1.06–1.56)^c^	1.66 (1.32–2.10)^c^
BMI (ref: healthy weight. BMI < 25)			
Overweight		0.80 (0.67–0.96)^c^	0.85 (0.71–1.02)
Obesity		0.94 (0.74–1.18)	0.99 (0.79–1.25)
Sleep [ref: average sleep (7–8 h)]			
Short sleep (<7 h)		0.89 (0.76–1.04)	0.81 (0.64–1.02)
Long sleep (>8 h)		1.77 (1.39–2.26)^c^	1.74 (1.25–2.42)^c^
Physical functioning (ref: good)			
Poor		1.07 (0.93–1.24)	1.05 (0.84–1.32)
Mental functioning (ref: good)			
Poor		1.23 (1.06–1.42)^c^	1.39 (1.15–1.68)^c^
Current pain (ref: no current pain)			
Yes		0.95 (0.84–1.08)	0.94 (0.78–1.14)

a Model 1 = age + gender; Model 2 = age + gender + each covariate independently; Model 3 = all covariates combined.

b RR for belonging to Trajectory 2 compared to Trajectory 1. Trajectory 1 = ‘Stable regular working hours’; Trajectory 2 = ‘Shorter and varying working hours’ (RR, relative risk; 95% CI, 95% confidence interval).

c Statistically significant.

In Model 2 (table 2), all the other covariates were modelled with age and gender independently. Compared to manual workers, professionals and managers were more likely (RR 1.87, 95% CI 1.54–2.27) to belong to the trajectory of ‘Shorter and varying working hours’. People who reported binge drinker (RR 0.82, 95% CI 0.71–0.95) were less likely and those who reported non-drinker more likely (RR 1.12, 95% CI 1.06–1.56) to belong to the trajectory of ‘Shorter and varying working hours’. People who reported BMI overweight but not obese (mean BMI 25–30) were less likely (RR 0.80, 95% CI 0.67–0.96) to belong to ‘Shorter and varying working hours’. Compared to those reporting sleeping 7–8 h, those reporting sleeping >8 h were more likely (RR 1.77, 95% CI 1.39–2.26) to belong to ‘Shorter and varying working hours’. Those reporting poor mental functioning belonged more often to ‘Shorter and varying working hours’ (RR 1.23, 95% CI 1.06–1.42) whereas there was no difference between trajectories in physical functioning.

In Model 3 (table 2), all covariates were simultaneously adjusted for. Women (RR 1.40, 95% CI 1.09–1.78) and the oldest age group (RR 2.71, 95% CI 2.06–3.57) had higher odds of belonging to the trajectory of ‘Shorter and varying working hours’. Professionals and managers were more likely (RR 1.56, 95% CI 1.20–2.02) and semi-professionals (RR 0.70, 95% CI 0.50–0.96) and routine non-manual workers (RR 0.74, 95% CI 0.55–0.98) less likely to belong to trajectory of ‘Shorter and varying working hours’. Non-drinkers (RR 1.66, 95% CI 1.32–2.10) and those reporting sleeping >8 h (RR 1.74, 95% CI 1.25–2.42) were more likely to belong to ‘Shorter and varying working hours’ whereas binge drinkers were less likely (RR 0.82, 95% CI 0.68–0.999). Those reporting poor mental health functioning had higher odds of belonging to ‘Shorter and varying working hours’ (RR 1.38, 95% CI 1.15–1.67). Marital status, smoking, mean BMI, physical functioning and current pain were unassociated with trajectory group membership.

## Discussion

This study examined the developmental trajectories of working hours during later work career over a period of nearly two decades. Social and health-related factors and their associations with the trajectories were examined. Two distinctive trajectories emerged: ‘Stable regular working hours’ (90%) and ‘Shorter and varying working hours’ (10%). Social determinants including female gender, age over 60 years, and higher occupational class were associated with a higher likelihood of belonging to the trajectory of ‘Shorter and varying working hours’. Health-related factors including reported sleeping >8 h, reporting non-drinker and poor mental functioning were associated with a higher likelihood of belonging to trajectory of ‘Shorter and varying working hours’ in the fully adjusted model. Smoking, BMI, physical functioning and current pain were unassociated with trajectory membership.

### Interpretation

To our knowledge, there are no studies that have examined trajectories of working hours from mid work career to later career and only few previous studies have examined working hours in later work careers.^4^^,^^16^^,^^17^^,^^28^ In our study, the majority of participants worked stable and regular working hours (defined as a maximum of 40 h per week), which is the most common limit of normal working hours.^29^ The participants assigned to the trajectory of ‘Stable regular working hours’ reported working hours at around 35–37 h per week, whereas the reported working hours in the smaller trajectory of ‘Shorter and varying working hours’ varied between 15 and 33 h per week. In our main analysis, no trajectory with reported working hours over 40 h per week was observed and the percentage of participants reporting over 40 h per week was small (7%, data not shown). The small amount of longer working hours is most likely affected by many factors. In the EU, the Directive mandates that 48 h per week is the maximum amount of weekly working hours, which includes overtime hours^30^ and working overtime is less prevalent in Nordic countries.^31^ In comparison, many Asian countries, including high-income countries like Singapore and the Republic of Korea are known for long working hours^29^ but these countries also have lower employment rates for women. The nature of our cohort might also affect the lack of reporting long working hours as municipal sector employees report less overtime work compared to the private sector and public sector employees are predominantly women. Our cohort also constituted of employees with permanent job contracts (>90%) excluding precarious job contracts.^32^

Women were more likely to belong to the trajectory with shorter working hours, which correlates with previous knowledge from Finland. Among women, shorter working hours might partly be explained due to requirements from home, such as taking care of their children, spouse or elderly parents but in Finland, women also report lack of full-time employment as a reason for part-time employment.

Older employees more often belonged to the shorter working hour trajectory. This is likely at least partly explained by the policies that allow older employees to reduce their working hours, such as part-time pension.^4^^,^^5^ In Finland, during our follow-up, employees could retire on part-time pension if certain criteria were met. On part-time pension, the employee has to cut down working hours and move to part-time employment. Part-time pension has not been widely utilized in Finland. For example, in 2016, 1.5% of employees eligible to part-time pension, retired to part-time pension.^33^

Partial disability pension also affects working hours. In case of illness and permanent loss of work ability employees could be granted part-time (or full-time) disability pension. After 60 years of age disability pension is granted on milder terms as work ability is evaluated only by employees’ potential to return to work in his current profession. Also relevant to our cohort is that pension age varies between occupations among municipal employees. During our study period, the Finnish earnings-related pension system underwent a reform in 2005 after which employees could choose to retire between 63 and 68 years of age. Also the part-time pension age limit was gradually lifted to 61 years. Later on, the part-time pension system was replaced by the partial old-age pension system in 2017.

Professionals and managers were also more likely to the shorter working hour trajectory. Occupational class has been associated differences in exiting workforce. Both those in the highest and lowest occupational classes are more likely to extend work career but for different reasons. For those in the lower occupational classes continuing working might be a financial necessity whereas those in more affluent position might have the chance to choose shorter work week or part-time-retirement regardless of their current health.^34^^,^^35^ Those in higher socio-economic position are also more likely continue working after retirement.^8^

From health-related factors only reported sleep, alcohol consumption and poor mental functioning were associated with trajectory membership. Epidemiological studies associate sleeping 7–8 h per night with good health. However, sleeping <7 h is common.^36^ In our study, a fifth of the participants reported sleeping <7 h and this was more common among members of ‘Stable regular working hours’ trajectory. Reporting sleeping more than 8 h per night was associated with higher likelihood of belonging to ‘Shorter and varying working hours’, which can indicate either that those working shorter hours have a better chance to sleep or that there are other factors affecting sleeping hours, which we were unable to take into consideration in our study setup. Reporting non-drinker was more common among members of ‘Shorter and varying working hours’ and reporting non-drinker was also more common among those reporting sleeping >8 h (*P* < 0.001, data not show). Alcohol consumption among other factors worsens the quality of sleep and binge drinking has been associated with insomnia symptoms.^37^ The above-mentioned findings warrant questions on whether alcohol consumption and sleep have a causal effect on the development of working hours.

Those reporting poor mental functioning more often belonged to trajectory of ‘Shorter and varying working hours’. In line with this a Belgian study found an association with working time reductions and a worsening of self-perceived health and depressive symptoms among employees above 50 years.^17^ There does not seem to exist a clear occupational class gradient in psychiatric diseases. At workplace physical demands have decreased while work has become increasingly mentally demanding. Physically strenuous work is considered in occupational health care but difficulties regarding psychosocially strenuous work might not be as well met. High job demands and low control have been associated with psychotropic medication among employees.^38^ Those in lower occupational classes might face low job control and have little possibilities to control their tasks during workdays whereas those in managerial occupations might suffer from higher job demands. Poor mental functioning emerging as one factor differing between the trajectories emphasizes employee mental health as one key factor in work ability.

We found no differences between the trajectories in physical functioning or current pain. However, we included only people who were employed part- or full-time. Being able to work is itself indicative of at least moderate health. Previous studies identify multiple different risk factors for chronic pain, such as lower socio-economic position^39^ and female gender.^40^

This study has multiple strengths. The longitudinal HHS data comprise a large employee cohort that includes a wide range information on sociodemographic factors, work arrangements, working conditions and health. Survey data do not allow confirming causal relationships but clear associations were observed. Additionally, the HHS data included only employees from a one municipal sector employer so caution should be used if generalizing these results into the general working population. Also, our data were collected at a 5-year interval. Having distant intervals might make smaller changes in working hours unnoticeable. Like any other statistical method, GMM and other finite mixture modelling methods are not flawless^27^ and represent an approximation. Still, this method enables studying the heterogeneity of the data and displaying longitudinal data in an understandable way.

In conclusion, our study showed developmental patterns in working hours from midlife towards the end of the working life. Being assigned to the shorter weekly working hour group was associated with social factors, such as gender and occupational class. Mental functioning, sleep and alcohol consumption emerged as possible health-related factors interacting with the development of working hours. More in-depth research on mental health and its connection to development of working hours is needed. Improving employees’ health behaviours and prevention of chronic diseases is likewise important as without good prevention and treatment of chronic diseases employees of all ages are at risk of disability.

## Supplementary data

Supplementary data are available at *EURPUB* online.

## Funding 

This work is supported by the Finnish Work Environment Fund (grant #117308 for T.L.), by the Academy of Finland (grant #1294514 for O.R.; grant #319200 for T.L.), by the Juho Vainio Foundation (for O.R.) and by the Finnish Medical Association (grant #3395 for J.S.-U.).

*Conflicts of interest*: None declared.

## Ethics approval

The ethics committees of the Department of Public Health, the University of Helsinki and the health authorities of the City of Helsinki have approved the HHS study.

## Patient consent for publication

Not required.

## Data availability statements

Data are available from Helsinki Health Study group upon reasonable request and following current data sharing policies.

**Key points**

- There are no previous studies looking at the development of working hours over time among employees in later careers.
- Two distinctive trajectories of working hours were identified: ‘Stable regular working hours’ (90%) and ‘Shorter and varying working hours’ (10%).
- Poor mental health, long sleep and non-drinking, high socio-economic position, female gender and older age emerged as determinants of the shorter working hours trajectory group.
- Promotion of mental well-being at workplace might facilitate continuing working normally in later careers.

## Supplementary Material

ckab179_Supplementary_Data
