# Supplementary material for: Trajectories of working hours in later careers and their association with social and health-related factors: a follow-up study
Source: Eur J Public Health. 2021 Oct 5;32(1):66–72. doi: 10.1093/eurpub/ckab179 (PMC8807068; doi:10.1093/eurpub/ckab179)
Supplement: ckab179_Supplementary_Data [file ckab179_supplementary_data.docx]

**Supplementary material**

**Supplementary table 1** Household net income* at baseline separately for men and women. Trajectory 1 = ‘Stable regular working hours’; Trajectory 2 = ‘Shorter and varying working hours’

|  |  | Trajectories | |  |
| --- | --- | --- | --- | --- |
|  |  | Trajectory 1: | Trajectory 2: | p value for chi^2^ |
|  | Study population | Stable normal working hours | Shorter and varying working hours |  |
| **Household income N (%)** |  |  |  |  |
| **Women** |  |  |  |  |
| 1. Quartile | 1765 (26%) | 1578 (26%) | 187 (27%)) |  |
| 2. Quartile | 1981 (29%) | 1795 (29%) | 186 (27%) |  |
| 3. Quartile | 1541 (23%) | 1398 (23%) | 143 (21%) |  |
| 4. Quartile | 1525 (22%) | 1347 (22%) | 178 (26%) |  |
|  | 6812 (100%) | 6118 (100%) | 694 (100%) | 0.007 |
|  |  |  |  |  |
| **Household income N (%)** |  |  |  |  |
| **Men** |  |  |  |  |
| 1. Quartile | 482 (28%) | 440 (27%) | 42 (32%) |  |
| 2. Quartile | 298 (17%) | 275 (17%) | 23 (18%) |  |
| 3. Quartile | 537 (31%) | 495 (31%) | 42 (32%) |  |
| 4. Quartile | 421 (24%) | 397 (25%) | 24 (18%) |  |
|  | 1738 (100%) | 1607 (100%) | 131 (100%) | 0.007 |

*Income was divided into 7 levels and weighted by household size^22^ and divided into quartiles. Additionally, information about work schedule was considered. For household income chi^2^ test was applied separately for men and women.

**Supplementary table 2**  R-code for calculating the selected model.

| model <- hlme(fixed = workinghours ~ poly(studyphase, degree = 2, raw = TRUE)*sex, random = ~ poly(studyphase, degree = 2, raw = TRUE), mixture = ~ poly(studyphase, degree = 2, raw = TRUE), subject= "ID", ng =numberoflatentclasses, data = datafile) |  |  |  |  |  |  |  |  |
| --- | --- | --- | --- | --- | --- | --- | --- | --- |

**Supplementary table 3** Criteria for determining the trajectory group membership

| Model | Number of latent classes | Number of parameters: | Maximum log-likelihood: | AIC: | BIC: | % class 1 | % class 2 | % class 3 | % class 4 |
| --- | --- | --- | --- | --- | --- | --- | --- | --- | --- |
| Ng1 | 1 | 13 | -66744 | 133514 | 133606 | 100.00 |  |  |  |
| Ng2 | 2 | 17 | -65166 | 130366 | 130487 | 9.68 | 90.32 |  |  |
| Ng3* | 3 | 21 | -64581 | 129204 | 129352 | 86.9 | 9.6 | 3.5 |  |
| Ng4* | 4 | 25 | -64657 | 129643 | 129542 | 86.9 | 3.5 | 9.6 | 0 |

* Model failed to converge

**Supplementary table 4** Posterior probabilities in each trajectory group

|  | ‘Stable regular working hours’ | ‘Shorter and varying working hours’ |
| --- | --- | --- |
| Number of participants assigned to trajectory group | 7941 | 851 |
| % | 90.32 | 9.68 |
|  |  |  |
| Mean of posterior probabilities in each class |  |  |
| Probability 1 | **0.997** | 0.055 |
| Probability 2 | 0.003 | **0.945** |
|  |  |  |
| Posterior probabilities above threshold (%) |  |  |
| probability > 0.7 | 99.72 | 98.94 |
| probability > 0.8 | 99.69 | 98.12 |
| **probability > 0.9** | **99.46** | **92.83** |

**Supplementary table 5** Social and health-related factors and their risk ratio (including household net income level) of belonging to Trajectory 2 compared to Trajectory 1 (Log-binomial regression model, risk ratio (RR for belonging to Trajectory 2 compared to Trajectory 1 and 95% CI)

|  | Model 1^a^ | Model 2 ^a^ | Model 3 ^a^ |
| --- | --- | --- | --- |
|  |  | RR (95%CI)b |  |
| Women | 1.41 (1.19–1.68)* |  | 1.37 (1.07–1.75)* |
| Age (ref: 40 years at phase 1) |  |  |  |
| Age 45 | 1.02 (0.81–1.28) |  | 1.04 (0.76–1.42) |
| Age 50 | 1.02 (0.81–1.28) |  | 1.01 (0.75–1.38) |
| Age 55 | 1.23 (0.99–1.52) |  | 1.12 (0.84–1.50) |
| Age 60 | 3.18 (2.61–3.88)* |  | 2.67 (2.02–3.53)* |
| Occupational class (ref: Manual workers) |  |  |  |
| Professionals and Managers |  | 1.87 (1.54–2.27)* | 1.66 (1.25–2.19)* |
| Routine non-manual workers |  | 0.86 (0.69–1.07) | 0.72 (0.54–0.97)* |
| Semi-professionals |  | 0.83 (0.65–1.07) | 0.72 (0.52–1.00) |
| Income (ref: Lowest quartile) |  |  |  |
| 2. Quartile |  |  | 0.79 (0.60–1.05) |
| 3. Quartile |  |  | 0.83 (0.64-1.07) |
| 4. Quartile |  |  | 0.78 (0.60–1.02) |
| Marital status (ref: Cohabiting) |  |  |  |
| Non-cohabiting |  | 0.96 (0.84–1.10) | 0.88 (0.71–1.09) |
| Health behaviour |  |  |  |
| Smoker (ref: Non-smoker) |  | 0.82 (0.69–0.98)* | 0.89 (0.68–1.17) |
| Drinking habits (ref: Non-binge drinker) |  |  |  |
| Binge drinker |  | 0.82 (0.71–0.95)* | 0.81 (0.67–0.99)* |
| Non-drinker |  | 1.28 (1.06–1.56)* | 1.61 (1.26–2.04)* |
| BMI (ref: Healthy weight. BMI<25) |  |  |  |
| Overweight |  | 0.80 (0.67–0.96)* | 0.83 (0.69–1.00) |
| Obesity |  | 0.94 (0.74–1.18) | 0.97 (0.77–1.23) |
| Sleep (ref: Average sleep (7–8h)) |  |  |  |
| Short sleep (<7h) |  | 0.89 (0.76–1.04) | 0.77 (0.61–0.98)* |
| Long sleep (>8h) |  | 1.77 (1.39–2.26)* | 1.70 (1.21–2.39)* |
| Physical functioning (ref: Good) |  |  |  |
| Poor |  | 1.07 (0.93–1.24) | 1.09 (0.87–1.36) |
| Mental functioning (ref: Good) |  |  |  |
| Poor |  | 1.23 (1.06–1.42)* | 1.38 (1.14–1.67)* |
| Current pain (ref: No current pain) |  |  |  |
| Yes |  | 0.95 (0.84–1.08) | 0.93 (0.77–1.12) |
|  |  |  |  |

^a^ Model 1 = age + gender; Model 2 = Age + gender + each covariate independently; Model 3 = All covariates combined

^b^ RR for belonging to Trajectory 2 compared to Trajectory 1. Trajectory 1 = ‘Stable regular working hours’; Trajectory 2 = ‘Shorter and varying working hours’ [RR= Relative risk, 95%CI= 95% confidence interval]
